# When the Going Gets Tough: The “Why” of Goal Striving Matters

**DOI:** 10.1111/jopy.12047

**Published:** 2013-08-07

**Authors:** Nikos Ntoumanis, Laura C Healy, Constantine Sedikides, Joan Duda, Brandon Stewart, Alison Smith, Johanna Bond

**Affiliations:** 1 University of Birmingham; 2 University of Southampton; 3 University of Bath; 4 University of Rochester

## Abstract

No prior research has examined how motivation for goal striving influences persistence in the face of increasing goal difficulty. This research examined the role of self-reported (Study 1) and primed (Study 2) autonomous and controlled motives in predicting objectively assessed persistence during the pursuit of an increasingly difficult goal. In Study 1, 100 British athletes (64 males; *M*_age_ = 19.89 years, *SD*_age_ = 2.43) pursued a goal of increasing difficulty on a cycle ergometer. In Study 2, 90 British athletes (43 males; *M*_age_ = 19.63 years, *SD*_age_ = 1.14) engaged in the same task, but their motivation was primed by asking them to observe a video of an actor describing her or his involvement in an unrelated study. In Study 1, self-reported autonomous goal motives predicted goal persistence via challenge appraisals and task-based coping. In contrast, controlled goal motives predicted threat appraisals and disengagement coping, which, in turn, was a negative predictor of persistence. In Study 2, primed autonomous (compared to controlled) goal motives predicted greater persistence, positive affect, and future interest for task engagement. The findings underscore the importance of autonomous motivation for behavioral investment in the face of increased goal difficulty.

Whether it is to perform well on an exam, to maintain physical health, or to stay ahead of the competition, goals form an integral part of daily life. A large literature has examined factors related to goal striving, such as how goals are activated (Fishbach & Ferguson, 2007), operate (Locke & Latham, 1990), are monitored (Zimmerman & Paulsen, 1995), and are guided by motives (Sheldon & Elliot, 1999). With regard to the last topic, the self-concordance (SC) model (Sheldon & Elliot, 1999) suggests that goal motives can be categorized as autonomous (based on personal interest, enjoyment, or perceived importance) or controlled (driven by internal or external pressures and contingencies related to social approval). Grounded in self-determination theory (SDT; Deci & Ryan, 1985, 2000), the SC model predicts that the more autonomous their motives are, the more individuals will sustain effort toward goal pursuit and eventually goal attainment. For example, Sheldon and Elliot (1998) and Sheldon and Houser-Marko (2001) demonstrated that autonomous reasons for the pursuit of academic goals related over time to goal attainment and well-being. Koestner, Lekes, Powers, and Chicoine (2002) also showed a positive relation between autonomous goal motivation and monthly progress on New Year's resolutions.

Although the advantages of autonomous motivation in mobilizing and allocating goal-related resources have been well documented (Sheldon, 2008), goal pursuit is rarely without its challenges. Indeed, achieving important life goals requires intense effort that is sustained over time in order to overcome difficulties and failures (Bandura, 1986; Dweck, 2007). Some goals are of fixed difficulty (e.g., achieving a certain grade in an academic exam), whereas others are of varying difficulty (e.g., keeping oneself in good physical condition). Although goal difficulty has been assessed in past research on goal striving, fluctuations in difficulty level over time, and how such fluctuations influence goal persistence, have largely been overlooked. For yet another category of goals, especially in achievement settings, difficulty can increase over time (e.g., staying ahead of the competition, being innovative). How individuals appraise and cope with increased goal difficulty, the implications for goal persistence and attainment, and the role of motivation for goal striving in this process have thus far escaped empirical attention in SDT/SC and the wider goal striving literatures. We address these issues in the context of sport. This is an achievement-driven environment, where the setting, pursuit, and regulation of goals are commonplace (Weinberg, Burke, & Jackson, 1997), and where perceptions of success are enhanced by evidence of triumph over mounting adversity (Goss, 1999). When striving to stay ahead of competition in sport, goal difficulty often increases over time (e.g., over a season or even within a competition; Johnson, 2011).

No prior research has examined the relation between motivation for goal striving and different levels of goal difficulty, that is, how motivation influences persistence in the face of increasing goal difficulty. Focusing on increased goal difficulty during striving is conceptually and empirically important. Levels of goal difficulty may differentially predict task performance depending on levels of goal commitment: Task performance is greatest under conditions of high goal difficulty and high goal commitment, as a meta-analysis by Klein, Wesson, Hollenbeck, and Alge (1999) found. This meta-analysis also reported that, of all personal antecedent variables examined, the strongest predictor of performance was personal volition (defined in terms compatible to personal autonomy: “a voice in the determination of the goal,” p. 890). This finding is consistent with an SC model/SDT perspective (Deci & Ryan, 1985; Sheldon & Elliot, 1999). When goal pursuit is fueled by personal endorsement and valuing of the goal, commitment and persistence will be high. In contrast, when goal pursuit is the outcome of pressures or external contingencies, commitment will always be “on the line” and goal attainment will be comparatively less likely. Indeed, in another meta-analysis pertaining to the SC literature, Koestner, Otis, Powers, Pelletier, and Gagnon (2002) obtained a moderate effect size between autonomous motivation and progress in goal pursuit (*d* = .42) and a negligible correlation (*d* = −.02) between controlled motivation and goal progress.

Previous research by Amiot, Gaudreau, and Blanchard (2004), Smith, Ntoumanis, Duda, and Vansteenkiste (2011), and a review by Gaudreau, Carraro, and Miranda (2012) have supported coping strategies as mediators of the relation between goal motivation and goal progress in sport and education. For example, Smith et al. (2011) reported that autonomous motivation predicted task-based coping, which in turn predicted goal effort and attainment. In contrast, controlled goal motives predicted disengagement coping, which in turn was a negative predictor of goal effort and attainment. These studies, however, were not experimental, and the measures of goal attainment were self-reported and, thus, possibly biased. Further, the studies did not examine the role of goal difficulty despite its documented influence on goal commitment and performance (Klein et al., 1999). In this article, we address these limitations using an experimental design with incremental goal difficulty and objectively measured goal attainment.

We conducted two studies to test the extent to which autonomous and controlled goal motives predict objectively assessed persistence toward challenging goals of increasing difficulty. Study 1 investigated personal motives toward a goal, whereas Study 2 primed goal motives. We hypothesized that autonomous (compared to controlled) motives would lead to greater persistence toward the goal.

## Study 1: Personal Motives and Persistence Toward an Increasingly Difficult Goal

Study 1 examined how goal motives impact on objectively assessed persistence when pursuing an increasingly difficult goal. We expected that, as autonomous motives reflect greater alignment with personal interests and values, individuals striving with these motives would initiate and sustain effort toward the goal, leading to greater persistence (Sheldon, 2008). Controlled goal motives may instigate positive intentions and efforts toward goal striving (Sheldon & Elliot, 1999); however, the motivational energy underpinning such motives is not linked to personal values. As such, striving with these motives is unlikely to lead to persistence, particularly in the face of difficulties.

Study 1 also examined whether the impact of goal motives on persistence is mediated by cognitive appraisals and coping strategies implicated in goal striving. We expected coping responses to goal challenges to influence the amount of persistence exerted (Lazarus, 1991). Previous research has indicated that task-based coping (e.g., increasing effort, relaxation) mediates the positive effects of autonomous motivation on self-reported goal effort and attainment, whereas disengagement coping (e.g., disengagement, venting of unpleasant emotions) mediates the negative effects of controlled motivation on goal effort and attainment (Amiot et al., 2004; Smith et al., 2011). The differing associations of goal motives with coping strategies and subsequent persistence may be further explained by cognitive appraisals of difficulties experienced in goal striving. In the transactional model of stress (Lazarus & Folkman, 1984), appraisals of stressful encounters precede coping responses. Autonomous motives may equip individuals to appraise goal difficulties as a challenge and thus adopt strategies to confront the difficulty; controlled motives may prompt individuals to appraise such difficulties as a threat, resulting in disengagement coping (Ntoumanis, Edmunds, & Duda, 2009; Skinner & Edge, 2002).

Integrating goal motives, cognitive appraisals, coping strategies, and behavioral persistence in the same model, we hypothesized that autonomous and controlled goal motives would be positively associated with challenge and threat appraisals, respectively. In turn, challenge and threat appraisals would, correspondingly, be positively related to task-oriented and disengagement-oriented coping strategies. Further, we hypothesized that task- and disengagement-oriented coping strategies would be positively and negatively linked to persistence, respectively. As goal striving is affected by perceived goal difficulty and goal efficacy (Locke & Latham, 2002), we controlled for these variables in the analyses.

## Method

### Participants

One hundred athletes (64 males, 36 females; *M*_age_ = 19.89 years, *SD*_age_ = 2.43) participated in exchange for course credit or financial reward (£5). These were recruited from the University of Birmingham and local sports clubs. The athletes were from a variety of team sports (except cycling or triathlon to avoid inclusion of participants with experience in cycling events of increasing difficulty) and trained on average 4.62 hours every week (*SD* = 2.45).

### Procedure

Participants completed the study individually in a single 1-hour session. They reported to the laboratory having avoided strenuous exercise for 24 hours, and also having avoided food, alcohol, caffeine, and tobacco for 3 hours prior. Participants were fitted with a heart rate (HR) monitor to record resting HR, before they responded to consent forms, a health screening questionnaire, and demographic questions. We developed the procedure following extensive pilot work. The procedure consisted of an incremental intensity exercise protocol on an electromagnetically braked cycle ergometer in hyperbolic mode, where power output is independent of pedal frequency. The main trial comprised 10 stages, each lasting 2 minutes. Participants were informed that we were investigating experiences of, and reactions to, success and failure when striving for a challenging goal, which was to complete all 10 stages of the trial. To complete a stage successfully and move on to the next, participants had to maintain at least 70 revolutions per minute (rpm) for the whole stage. Participants were aware that, if their intensity dropped below 70 rpm for a period of longer than 5 seconds, the trial would cease; they could voluntarily withdraw at any point. The intensity (resistance) of each stage was based on a percentage of mean power output (to control for differences in fitness and gender), determined via a 3-minute maximal output test completed prior to the main trial. The intensity increased from one stage to the next; thus, the goal of successful stage completion became increasingly difficult during the trial. During a 10-minute rest period before the main trial, participants completed goal-related measures. After the trial, they rated their cognitive appraisals and coping strategies during the trial. Debriefing concluded the laboratory session.

### Measures

#### Goal-Related Variables

To assess goal motivation, participants rated four items used previously in goal striving research (Sheldon & Elliot, 1999; Smith, Ntoumanis, & Duda, 2007; Smith et al., 2011). Specifically, they rated (1 = *not at all*, 7 = *very much so*) the extent to which they were pursuing the goal of completing all 10 stages for extrinsic (“because you feel you are expected to do so”), introjected (“because you would feel embarrassed or anxious if you didn't”), identified (“because the goal will give you personally important information”), or intrinsic (“because of the enjoyment or challenge the pursuit of the goal provides you”) reasons. Aligned with SC model research, we created an autonomous motives score by averaging the scores for the identified and intrinsic motivation items. Likewise, we formed the controlled motives score using the mean response to the extrinsic and introjected items. Participants also responded to three goal difficulty (e.g., “how difficult is your goal?”) and three goal efficacy (e.g., “how strong is your belief that you are able to achieve your goal?”) items (1 = *not at all*, 7 = *very much so*).

#### Cognitive Appraisals

Following the trial, participants completed five items for both challenge (e.g., “I viewed the task as a positive challenge”) and threat (e.g., “I thought the task could have been threatening to me”) appraisals of the task (1 = *not at all true of me*, 7 = *very true of me*). We adapted these items from research on academic goals (McGregor & Elliot, 2002).

#### Coping Strategies

We measured task-based coping (e.g., “I gave my best effort”) with three items from the effort expenditure scale of the English version of the Inventaire des Strategies de Coping en Compétition Sportive (ISCCS; Gaudreau & Blondin, 2002), and with one item from the Active Coping scale of the COPE (Carver, Scheier, & Weintraub, 1989). We measured disengagement coping (e.g., “I let myself feel hopeless and discouraged”) with four items from the Disengagement/Resignation scale of the ISCCS. Responses ranged from 1 (*not at all*) to 7 (*very much so*).

#### Persistence

We operationalized persistence primarily as the total number of stages completed. We also measured HR at the end of the trial as a supplementary indicator of persistence. We measured resting HR prior to the study and at every stage of the main trial. HR increases during exercise can be used as an indicator of the central command system, which is related to the parallel activation of cardiovascular and motor systems during exercise and the individual's perception of the effort required to perform a task (Williamson, Fadel, & Mitchell, 2006). As such, working at higher HR levels could indicate that the individual is exhibiting higher persistence toward his or her goal. An individual's maximum HR will vary with age and can be predicted by subtracting age from 220; therefore, to standardize the variable across all participants, we expressed the HR that participants reached when they ceased the trial as a percentage of their age-predicted maximum. We also took into account participants' resting HR in order to control for baseline differences that may have been due to genetic or fitness factors.

### Results

#### Descriptive Statistics, Scale Reliabilities, and Pearson's Correlations

Table 1 displays descriptive statistics, scale reliabilities, and Pearson's correlations for the variables of the hypothesized model. Most variables exhibited satisfactory internal reliability, with the exception of the autonomous and controlled goal motives scales, which had lower reliability coefficients (probably because each scale comprised only two items). Both scales, however, had significant inter-item correlations (.45 and.48 for autonomous and controlled motives, respectively). Given that the items for goal motives reflected adjacent motivational regulations along the SDT continuum and not the same motivational regulation, we consider the correlations satisfactory. Nevertheless, in our analysis we ensured that the paths in the hypothesized model were not attenuated by measurement error (see below). Consistent with previous research (Smith et al., 2007), autonomous and controlled goal motives were unrelated, supporting their treatment as separate factors in the hypothesized model. However, contrary to other SC literature (Smith et al., 2007, 2011; Smith, Ntoumanis, & Duda, 2010), the mean ratings for autonomous and controlled goal motives were very similar (4.37 and 4.35, respectively). This could be explained by the novelty of the trial; in previous studies, participants rated motives for familiar goals. The mean level of persistence was just over five stages completed, suggesting that our pilot work had been successful in designing a task that was difficult but achievable, while allowing for variations in motivation factors related to the goal.

**Table 1:** Study 1: Descriptive Statistics, Internal Reliabilities, and Pearson's Correlations Among Variables

	*M*	*SD*	α	1	2	3	4	5	6	7
1. Autonomous motives	4.37	1.28	.62	—						
2. Controlled motives	4.35	1.30	.63	−.10	—					
3. Challenge appraisals	4.88	1.04	.87	.54**	.02	—				
4. Threat appraisals	2.91	1.09	.80	−.06	.46**	−.04	—			
5. Task coping	5.14	1.00	.84	.46**	.01	.64**	−.09	—		
6. Disengagement coping	3.13	1.19	.77	−.24*	.30**	−.38**	.39**	−.30**	—	
7. Persistence/stages completed	5.01	1.58	—	.39**	.08	.38**	.05	.30**	−.30**	—
8. Persistence/percentage max HR	90.98	4.88	—	−.07	.18	.27**	.11	.29**	−.14	.20*

*Note*. HR = heart rate.

*p < .05. **p < .01.

#### Goal Motives, Appraisals, Coping Strategies, and Persistence

We tested the hypothesized model with structural equation modeling (SEM), using EQS 6.1 (Bentler, 2003), and specifying a robust maximum likelihood estimation method. Each latent factor had one indicator, representing the mean score for all items reflecting that factor. Single-indicator latent factor models are particularly suited for a sample size insufficient for a multiple-indicator SEM. In single-indicator models, measurement error can be incorporated in the analyses (as with multiple-indicator models), and thus the parameters of the structural model are not attenuated by measurement error (Hayduk, 1987). Given that preliminary analyses showed significant gender differences in the mean scores in cognitive appraisals and effort coping (i.e., males scored significantly higher on challenge appraisals and effort coping, and lower on threat appraisals, than females), we controlled for gender in the analyses. First, we tested a model with autonomous and controlled motives predicting persistence. The model fit the data well: χ^2^(4) = 2.38, *p* = .66, CFI = 1, NNFI = 1, SRMR = .05, RMSEA = 0. Autonomous motives predicted persistence (β = .50, *p* < .001), but the path from controlled motives was not significant (β = .15, *p* = .18).

**Figure 1:**
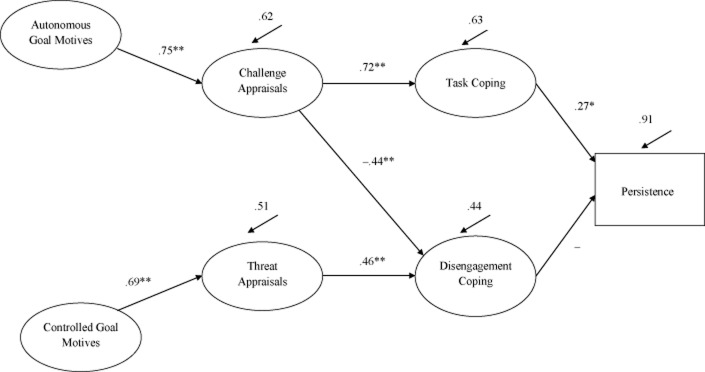
Study 1: Model showing the relation between goal motives, cognitive appraisals, coping, and persistence. The measurement model is omitted for presentation simplicity reasons. *p < .05. **p < .01.

Next, we tested the hypothesized single-step multiple-mediator model (Figure 1; Preacher & Hayes, 2008), including appraisals and coping strategies. This model fit the data relatively well: χ^2^(19) = 39.56, *p* = .003, CFI = .99, NNFI = .98, SRMR = .11, RMSEA = .11, but the modification indices indicated the addition of a negative path from challenge appraisals to disengagement coping. This path was conceptually appropriate (Ntoumanis et al., 2009), and we thus added it to the model, which showed improved fit: χ^2^(17) = 22.62, *p* = .16, CFI = .97, NNFI = .95, SRMR = .06, RMSEA = .06. The direct effects in Figure 1 indicate that autonomous motives were strong positive predictors of challenge appraisals, which in turn strongly predicted effort coping. In contrast, controlled goal motives were strong predictors of threat appraisals, which predicted disengagement coping. In addition, challenge appraisals negatively predicted disengagement coping. The number of stages completed was predicted positively by effort coping and negatively by disengagement coping. In line with the recommendations of Preacher and Hayes (2008), bias-corrected bootstrapped 95% confidence intervals (BC-CI) were also calculated for the indirect effects. We obtained significant indirect effects on persistence from autonomous goal motives (β = .22, *p* < .01, BC-CI = .08 to.36) and challenge appraisals (β = .30, *p* < .001, BC-CI = .15 to.44). The indirect effects of controlled motives (β = −.07, *p* = .08, BC-CI = −.15 to.01) and threat appraisals (β = −.10, *p* = .07, BC-CI = −.22 to.01) on persistence were not significant.

The structural paths in Figure 1 (as well as in the initial model that included goal motives and persistence only) remained significant and largely unchanged when we added goal difficulty, goal efficacy, and hours of sport training per week (in addition to gender) as control variables. Additional multiple regression analyses further bolstered the validity of these findings by showing that, after controlling for resting HR, autonomous goal motives positively predicted (β = .21, *p* = .04) the percentage of maximum HR reached by participants when they ceased the trial. The effect of controlled goal motives was not significant (β = −.01, *p* = .89).

### Discussion

The results of Study 1 indicate that autonomous motives are associated with an adaptive self-regulatory response (i.e., greater persistence) for a goal that becomes increasingly difficult to attain. This motivation for goal striving based on enjoyment or personal importance can lead to greater behavioral investment in goal pursuit. In contrast, controlled goal motives, which are based on meeting others' expectations or avoiding embarrassment, were not associated with any of these indicators of behavioral investment. Study 1 also showed that cognitive appraisals and coping responses to goal challenges partly explained the impact of goal motivation on goal-related persistence (Lazarus, 1991). When faced with an increasingly difficult goal, individuals with high autonomous goal motivation view the situation as a challenge, use task-focused coping, and display increased persistence. Autonomous goals are self-endorsed; therefore, goal challenges are seen as opportunities for personal mastery and not as threats to self-worth (Smith et al., 2011). Individuals with controlled goal motivation, however, view the same situation as threatening and display disengagement-based coping, as well as decreased persistence (unless they are ego involved, in which case they might display a short-lived persistence; Ryan, Koestner, & Deci, 1991). This is probably because, when goals are regulated by controlled motives, the internal conflicts or external pressures associated with such motivation can be mentally draining and energy consuming, thus resulting in fewer resources available to persist when faced with goal difficulties (Moller, Deci, & Ryan, 2006). Such an explanation could also account for the nonsignificant indirect effects of controlled motives and threat appraisals on persistence. Our findings support relevant theorizing (Ntoumanis et al., 2009; Skinner & Edge, 2002) and illustrate some of the means through which striving with autonomous motivation leads to greater persistence in pursuit of an increasingly difficult goal.

Study 1 adds to the goal striving literature by illustrating the role of personal goal motives in predicting persistence with an increasingly difficult goal. This study, however, did not examine how motivation for goal pursuit can be primed by external factors or the aftermath of persistence. Study 2 addressed this limitation by creating autonomous supportive, controlling, and neutral motivational primes. This follow-up study examined how primed motivation affects not only persistence, but also additional cognitive and affective outcomes.

## Study 2: Primed Motives and Persistence Toward an Increasingly Difficult Goal

Study 2 had three objectives. The first was to replicate the Study 1 finding that autonomous (compared to controlled) goal motives instigate greater persistence when striving for an increasingly difficult goal. The second objective was to examine the impact of autonomous (vs. controlled) goal striving and persistence on cognitive and affective outcomes variables. The third and final objective was to examine the effects of priming goal motivation. Previous SDT-based work has primed general motivational tendencies for autonomous and controlled motivation, but it has not primed motivation for pursuing a particular goal (Friedman, Deci, Elliot, Moller, & Aarts, 2010; Hodgins, Yacko, & Gottlieb, 2006; Levesque, Copeland, & Sutcliffe, 2008; Radel, Sarrazin, & Pelletier, 2009; Ratelle, Baldwin, & Vallerand, 2005).

Instead of exploring mediators of the relation between motives and persistence (as in Study 1), Study 2 examined potential outcomes of goal persistence, namely, changes in positive affect and interest in future goal engagement. Based on previous cross-sectional and longitudinal goal striving research (Sheldon & Elliot, 1999; Smith et al., 2007, 2011), we expected that successful goal pursuit (as facilitated by more autonomous strivings) would lead to greater outcomes for psychological well-being (i.e., positive affect). Furthermore, we anticipated that the greater levels of positive affect would lead to interest in future goal engagement, given previous findings suggesting that positive affect can motivate individuals to invest time and effort into their goals (Haase, Poulin, & Heckhausen, 2012). Also, as autonomous goal pursuit is regulated through interest (Ryan & Deci, 2000), we expected autonomous motivation to lead not only to increased goal persistence, but also directly to interest in future goal engagement.

There have been various priming techniques employed in the SDT literature, such as sentence scrambling (Hodgins et al., 2006) and subliminal word priming (Radel et al., 2009). However, it is difficult to translate these methods to a sporting environment, as they would not occur naturally in training or competition. As such, it is possible that previous priming techniques lack ecological validity for sport-based research. A notable exception is work by Friedman et al. (2010), in which participants' motivational orientation was successfully primed using a confederate who appeared to be motivated in either an autonomous or a controlled manner. We considered the use of a confederate; however, this was impractical in the present context and thus we instead used a video. Video use ensures that the prime is consistent across participants. This technique is considered to be a mind-set prime (Bargh & Chartrand, 2000; Gollwitzer, 1990), whereby participants are exposed to a goal-directed thought (i.e., the motivation for a goal, in the present study), which is more likely to operate subsequently in a different, unrelated context. Furthermore, while athletes often speak anecdotally of how their motivation can be influenced by role models they see on television, this possibility has not been explored by sports motivation research. Essentially, we aimed to prime a contextual factor to influence an athlete's motivation for his or her goal. In all, we primed autonomous and controlled motivation as well as a neutral condition with no motivational content. We included the neutral condition to be able to compare across conditions both the positive effects of autonomous motives and the negative effects of controlled motives on goal pursuit.

In summary, we were concerned in Study 2 with the impact of priming goal motivation on persistence toward an increasingly difficult goal. We hypothesized that primed autonomous and neutral goal motives would lead to greater persistence toward a goal in a cycling task compared to primed controlled motives. Furthermore, we hypothesized that persistence would positively predict both positive affect and future interest. In addition, we expected future interest, but not positive affect, to be directly predicted by the autonomous prime, as interest is the driving force in autonomous goal motives (Ryan & Deci, 2000), and positive affect (being an aspect of subjective well-being) is more likely to be an outcome of persistence and accomplishment (Sheldon & Elliot, 1999). Finally, we hypothesized that persistence would predict future interest indirectly through positive affect.

## Method

### Participants

Ninety athletes (47 female, 43 male; *M*_age_ = 19.63 years, *SD*_age_ = 1.14) from various sports (except cycling and triathlon) participated for course credit or financial reward (£5). These athletes were recruited in the same manner as in Study 1 and trained on average 4.58 hours per week (*SD* = 2.91).

### Procedure

We used a protocol similar to Study 1, with the exception that participants were randomly assigned to a priming condition (autonomous, controlled, neutral). We presented the prime to participants on a computer screen immediately before the main exercise trial. Participants observed a video of an actor describing her or his upcoming involvement in a study. We matched actor and participant gender. As a cover story, participants were told that the video was from an unrelated study investigating exercise and memory, and that they would be asked video-relevant questions following the main trial. We developed the actor scripts to describe a task that involved working toward a goal, but also to reflect the different goal motives. The autonomous motives prime portrayed challenge, the gain of personally important information from task engagement, and the feeling that the goal would be difficult but enjoyable. In contrast, the controlled motives prime portrayed perceived pressure and goal striving resulting from feelings of guilt. The neutral prime contained no motivational or goal pursuit content; the actor simply described the task used in an (unpublished) imagery effectiveness study, which constituted the second author's master's thesis (Healy, Roberts, & Hardy, 2009).

For manipulation checks, participants rated the extent to which the actor was striving with autonomous (i.e., “expected to enjoy the activity they were about to do,” “felt the activity in their trial was personally important to them”) and controlled (i.e., “were going to try and achieve their goal to avoid feeling guilty,” “were completing the activity because of a research hour or payment”) motives on a 1 (*not at all*) to 7 (*very much so*) scale. These items were presented as memory questions to support the cover story given to participants. To maintain the pretense and effectiveness of the prime, we asked these questions after the main trial, as research has suggested that presenting such items immediately after the prime can invoke suspicion and lessen the impact of the prime on the desired outcome behavior (Strack, Schwarz, Bless, Kubler, & Wanke, 1993). We collected outcome measures (see below) and implemented funneled debriefing (Bargh & Chartrand, 2000) after the main trial.

### Measures

#### Positive Affect

On arrival and after the main trial, participants rated (1 = *do not feel*, 5 = *feel very strongly*) how they felt “right now” on the Positive Engagement subscale of the Exercise-Induced Feeling Inventory (EFI; Gauvin & Rejeski, 1993). This subscale comprises three items: “enthusiastic,” “happy,” and “upbeat.”

#### Future Interest

Following the main trial, we used a three-item measure (i.e., “I would be interested in participating in this study again in the future”; “I would recommend this study to my friends”; “I would be interested in participating in other studies like this one in the future”) to assess interest in future participation in the same or similar studies (1 = *not at all*, 7 = *very much so*). We generated these items for the purpose of this study.

#### Control Variables

Similar to Study 1, we assessed goal difficulty and efficacy as control variables.

#### Persistence

As in Study 1, we operationalized persistence as the total number of stages completed and as the percentage of age-predicted maximum HR achieved at the end of the trial, controlling for baseline HR.

## Results

### Preliminary Analyses

**Figure 2:**
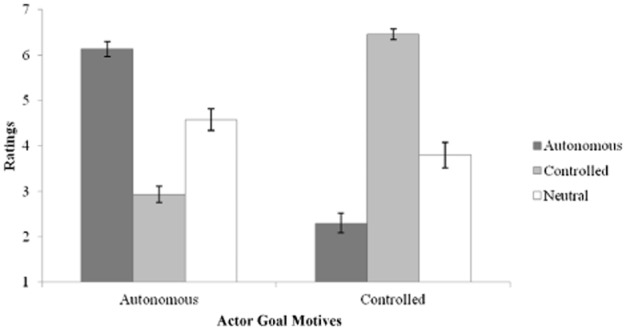
Ratings of actor's goal motives across priming conditions. All means significantly different at *p* < .001.

We removed six participants (one had previous triathlon experience; five indicated suspicion of the prime) from all analyses, leaving data from 84 participants (43 female; *M*_age_ = 19.58 years, *SD*_age_ = 1.12). Three ANOVAs revealed that participants in the three primed groups (27 autonomous, 27 controlled prime, 30 neutral) did not differ in age or in number of hours spent training or cycling per week, *F*(2, 81) < 1.82, *p* > .05, partial η^2^ = .04. Furthermore, a MANOVA showed that the manipulation was successful: Pillai's Δ = .78, *F*(4, 162) = 26.07, *p* < .001, partial η^2^ = .39 (Figure 2). Specifically, participants rated the actor as having stronger autonomous motives in the autonomous prime (*M* = 6.15, *SD* = .82) than in the controlled (*M* = 2.93, *SD* = .95) and neutral (*M* = 4.58, *SD* = 1.32) primes, *F*(2, 81) = 62.20, *p* < .001, partial η^2^ = .60. Conversely, participants rated the actor as having stronger controlled goal motivation in the controlled prime (*M* = 6.46, *SD* = .65) than in the autonomous (*M* = 2.30, *SD* = 1.16) and neutral (*M* = 3.80, *SD* = 1.51) primes, *F*(2, 81) = 86.79, *p* < .001, partial η^2^ = .68. We display these findings in Figure 2.

### Descriptive Statistics, Scale Reliabilities, and Pearson's Correlations

We present descriptive statistics, scale reliabilities, and Pearson's correlations in Table 2. All scales showed an appropriate level of internal reliability (αs > .70). Participants reported higher positive affect when they arrived at the laboratory than after the main trial, probably due to the physical investment in this trial and the associated exertion. We created a residual score for this variable and used it in the SEM analysis. We employed nonorthogonal contrast coding to compare the effects of the primes. We were interested in the difference between autonomous versus controlled motivation on persistence, and whether controlled motivation undermined persistence compared to a “no prime” motivation condition. Thus, we used the controlled prime as the reference category to create autonomous versus controlled and neutral versus controlled contrasts, which became independent variables in subsequent analyses. The descriptive statistics and internal reliabilities for the outcome variables across the prime conditions are presented in Table 3.

**Table 2:** Study 2: Descriptive Statistics, Internal Reliabilities, and Pearson's Correlations Among Variables

	*M*	*SD*	α	1	2	3	4	5
1. Pre positive affect	3.10	.68	.70	—				
2. Autonomous vs. controlled contrast	—	—	—	−.02	—			
3. Neutral vs. controlled contrast	—	—	—	.04	−.51**	—		
4. Persistence	4.10	1.44	—	−.10	.24*	.07	—	
5. Post positive affect	2.75	.79	.70	.25*	.03	.02	.31**	—
6. Future interest	4.65	1.35	.85	.20	.23*	−.03	.23*	.40**

*Note*. *p < .05. **p < .01.

**Table 3:** Study 2: Descriptive Statistics and Internal Reliabilities for Positive Affect, Persistence, and Future Interest Across the Prime Conditions

	Autonomous Prime			Controlled Prime			Neutral Prime		
	*M*	*SD*	α	*M*	*SD*	α	*M*	*SD*	α
Pre positive affect	3.09	.75	.76	3.07	.68	.71	3.14	.64	.63
Persistence	4.59	1.39	—	3.44	1.34	—	4.23	1.38	—
Post positive affect	2.78	.83	.73	2.70	.79	.61	2.76	.76	.75
Future interest	5.10	1.13	.81	4.27	1.40	.82	4.58	1.40	.87

### Primed Goal Motives, Persistence, Positive Affect, and Future Interest

**Figure 3:**
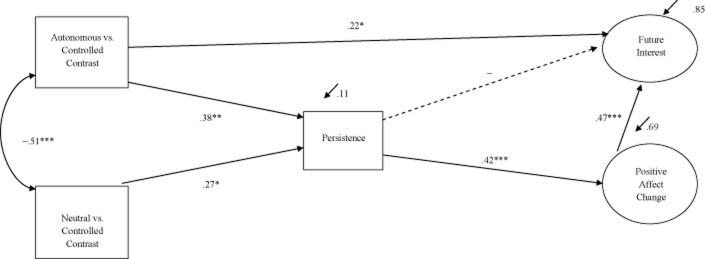
Model showing the relation between contrasts of primed motives, persistence, positive affect change, and future interest. *p < .05. **p < .01. ***p < .001.

We tested the hypothesized model with SEM, utilizing the single-indicator approach described in Study 1. This model showed excellent fit: χ^2^(4) = 1.35, *p* = .85, CFI = 1, NNFI = 1.19, RMSEA = .00, SRMR = .02 (Figure 3). Both the autonomous versus controlled (β = .38, *p* < .01) and the neutral versus controlled (β = .27, *p* = .02) contrasts predicted persistence, although the latter effect was possibly due to suppression (see the correlation between the neutral vs. controlled contrast and persistence reported in Table 2). Persistence predicted positive affect change (β = .42, *p* < .01), which consequently led to greater interest in future study participation (β = .47, *p* < .01). The hypothesized pathway from the autonomous versus controlled contrast to future interest was significant (β = .22, *p* = .02), but the pathway from persistence to future interest was not significant (β = −.07, *p* = .95). We obtained, however, an indirect effect of persistence on future interest via positive affect change (β = .20, *p* < .01, BC-CI = .07 to.32). We also obtained significant indirect effects from the autonomous versus controlled contrast (β = .16, *p* = .01, BC-CI = .09 to.49), and marginal effects for the neutral versus controlled contrast (β = .11, *p* = .06, BC-CI = .04 to.46) on positive affect change through persistence. In an exploratory analysis, we specified a pathway from the neutral versus controlled contrast to future interest; this was not significant (β = .10, *p* = .45) and had minimal impact on the model fit. The model remained unchanged when we added gender, hours of cycling, hours of training, goal difficulty, and efficacy as control variables. We depict the final model in Figure 3.

In line with Study 1, we conducted additional multiple regression analyses. However, when controlling for resting HR, neither the autonomous versus controlled contrast (β = .15, *p* = .25) nor the neutral versus controlled contrast (β = .05, *p* = .69) predicted the final percentage of maximum HR reached by participants.

## Discussion

Study 2 shows that external motivational cues can influence task engagement when pursuing an increasingly difficult goal. We successfully primed different motivational factors using a procedure that is practical and ecologically sound for sport research. Study 2 supports and extends the Study 1 results by demonstrating that primed autonomous goal motives can impact upon persistence toward an increasingly difficult goal. Furthermore, the benefits of striving with autonomous motives extend further than behavioral investment to changes in positive affect, consistent with previous goal striving research (Sheldon & Elliot, 1999; Smith et al., 2007, 2011). Moreover, Study 2 advanced past literature by showing that autonomous motives can lead to enhanced interest in future goal engagement. Persistence also leads to greater future interest, albeit indirectly through positive affect change. These findings demonstrate the benefits of striving with autonomous motives, not only for goal pursuit, but also for affective outcomes and future goal engagement, which could encourage continued persistence.

The neutral prime, when compared with the controlled motives prime, resulted in greater persistence. This result is somewhat contradictory to other work (Hodgins et al., 2006), which reported that an impersonal prime produced worse performance than both an autonomous and a controlled prime; however, it is possible that this path was due to a suppression effect, as the relation between these two variables at the bivariate level was nonsignificant. Also, in Study 2, goal motives did not predict additional measures of persistence (e.g., HR) as in Study 1. However, the statistical relations (despite being nonsignificant in Study 2) were in the same direction and were not substantially different in magnitude across the two studies. It is possible that the nonsignificant findings in Study 2 were due to having a dichotomous predictor (i.e., the two prime contrasts) rather than a continuous variable as in Study 1.

## General Discussion

Literature on the SC model has shown that autonomous (compared to controlled) motives lead to greater goal attainment and more positive affective outcomes (Sheldon & Elliot, 1999; Smith et al., 2011). However, no previous work has examined the role of motivation for goal striving when faced with increasing goal difficulty. The present research complemented and extended previous investigations while supporting the central hypothesis that autonomous goal motives will result in greater objectively assessed persistence toward an increasingly difficult goal. These findings further illustrate the benefits of autonomous motives for adaptive goal regulation; if individuals strive with more autonomous motives, they will be better equipped to overcome challenges in goal pursuit.

As well as being the first research to examine the role of motives for a goal with increasing difficulty, the two studies extended the SC model (Sheldon & Elliot, 1999) by exploring the mediators and outcomes of the relation between goal motives and persistence. Study 1 demonstrated that the relation between personal autonomous motives and persistence is mediated by adaptive appraisals and coping. Study 2 showed that increased persistence, as a result of primed autonomous motives, leads to positive outcomes such as higher positive affect and stronger interest in future task engagement. In all, the current research adds to knowledge about how autonomous and controlled motivation produce variations in patterns of goal striving in achievement settings, using a combination of self-reports and objective measures.

The use of the prime in Study 2 presents a significant improvement on prior goal striving research. To the best of our knowledge, this was the first example of motivation for a specific goal being manipulated and being shown to have an effect on persistence and future interest. Furthermore, we used a prime that was not only successful in manipulating goal motivation, but also ecologically valid and easily applicable to a variety of real-world settings. This is a clear advantage over other priming techniques (e.g., sentence scrambling, subliminal priming) used in motivation research (Hodgins et al., 2006; Radel et al., 2009), while also being more practical than the involvement of a confederate (Friedman et al., 2010).

The sport setting that we implemented allowed us to objectively assess persistence and to manipulate goal difficulty in the same manner for all participants. However, the wider processes tested in the two structural equation models; the measures of goal motivation, coping, appraisals, affect, and goal interest; the primes that we used; and the empirical findings are of wider relevance and offer vital information for other achievement settings, such as business and education. In fact, our results are broadly aligned with similar work in other contexts (Koestner et al., 2002) regarding the beneficial role of goal striving with autonomous motives. Given that individuals are faced with increased goal difficultly when pursing important goals in various life domains (Bandura, 1986; Dweck, 2007), our work reinforces calls for developing social environments that facilitate such motives (Smith et al., 2011).

## Limitations and Future Research Directions

A potential limitation of our research is the relatively low internal reliability of the goal motive measures in Study 1. Although these are below conventional levels of reliability, there may be methodological and conceptual explanations. To begin with, autonomous and controlled motives only contained two items each, making it more difficult to obtain a high Cronbach's alpha. Furthermore, the individual items for each motive did not represent exactly the same facet of autonomous or controlled motivation. Items that were aggregated for autonomous motives reflected intrinsic and identified motivation, whereas the controlled items assessed extrinsic and introjected motives. While undoubtedly related, these are, for the most part, separate motives (Deci & Ryan, 1985, 2000; Sheldon & Elliot, 1999).

We demonstrated that autonomous motives are advantageous for an increasingly difficult goal. However, we are not aware of any studies that have directly compared the role of goal motivation when pursuing goals of varying difficulty (e.g., low, moderate, or highly difficult throughout, or randomly difficult). We suspect that under conditions of low or moderate goal difficulty, the relations between autonomous and controlled goal motives with persistence would not be so different as those found in our research. Indeed, Sheldon and Elliot (1998) suggested that controlled goal motives can predict initial effort toward goals; however, this effort is unlikely to be maintained when encountering challenges in goal pursuit. Hence, when challenges are not experienced, the initial efforts of those with controlled motives may be sufficient to result in goal attainment. However, under conditions of random or high difficulty, the relations we obtained in this research might replicate or be even stronger. Future work will do well to compare the role of autonomous and controlled motivation when pursuing goals of varying difficulty levels.

A further venture for future work would be to explore the interactions between an individual's personal motives and a situational prime, and how these may impact on adaptive goal regulation. In a further effort to link concepts from the SC and self-regulation literatures, future investigations will do well to also explore the role of goal motives in relation to unfulfilled goals and multiple goal striving. Recent findings have substantiated the negative impact of unfulfilled goals on subsequent performance in other tasks (Masicampo & Baumeister, 2011a, 2011b). It is worth testing whether the motivation for goal striving can moderate such responses to goal failure. Based on the SC literature, we hypothesize that individuals with autonomous (vs. controlled) goal motives will respond with more adaptive behavior to goal failure and also that their subsequent performance will not be compromised by the preceding failure. There are additional indicators of psychological well-being (or ill-being) that could be explored other than affect, such as subjective vitality, depression, or burnout. Furthermore, goal motives research has exclusively looked at motives toward a single goal. Individuals, however, frequently pursue multiple goals concurrently (Louro, Pieters, & Zeelenberg, 2007). Thus, it is pertinent to examine how goal motives impact upon effective goal striving when managing multiple goals, especially when the motivation across goals is incongruent (e.g., autonomous for one goal and controlled for another). In addition, future empirical efforts could determine factors that help individuals decide whether they should persist in their goal pursuit or strategically disengage from their goal and reengage in a different goal, given that disengagement, and not persistence, might be the adaptive self-regulatory response to goal difficulties under certain conditions (Wrosch, Miller, Scheier, & Pontet, 2007).

The findings of our research have implications for those striving in achievement settings, such as sport, business, and education. When individuals are engaging in goal setting, they will benefit from identifying goals that they enjoy or consider personally important. Such motivation can be beneficial, behaviorally and affectively, especially when goals become increasingly difficult over time. Practitioners who aim to facilitate effective goal setting in sport, business, and educational settings would benefit from Deci and Ryan's (2000) guidelines for developing autonomous motivation.

## Conclusion

To conclude, the present research supports and extends previous findings regarding the role of autonomous motivation for adaptive goal striving. Applications of these findings to sport settings could help athletes (and their coaches) be more effective in their goals. Regardless of whether motives are personal or externally primed, pursuing goals with autonomous motives sparks greater positive outcomes in terms of behavioral investment (both immediately and interest in future investment) and psychological well-being.

## References

[b1] Amiot CE, Gaudreau P, Blanchard CM (2004). Self-determination, coping, and goal attainment in sport. Journal of Sport & Exercise Psychology.

[b2] Bandura A (1986). Fearful expectations and avoidant actions as coeffects of perceived self-inefficacy. American Psychologist.

[b43] Bargh JA, Judd C, Chartrand TL, Reis H (2000). Studying the mind in the middle: A practical guide to priming and automaticity research. Handbook of research methods in social psychology.

[b9001] Bentler PM (2003). EQS 6.1 for Windows.

[b3] Carver CS, Scheier MF, Weintraub JK (1989). Assessing coping strategies—A theoretically based approach. Journal of Personality and Social Psychology.

[b4] Deci EL, Ryan RM (1985). Intrinsic motivation and self-determination in human behavior.

[b5] Deci EL, Ryan RM (2000). The “what” and “why” of goal pursuits: Human needs and the self-determination of behavior. Psychological Inquiry.

[b6] Dweck CS, Gordon S, Morris T, Terry P (2007). Self-theories: The mindset of a champion. Sport and exercise psychology: International perspectives.

[b7] Fishbach A, Higgins TE, Ferguson MF, Kruglanski AW (2007). The goal construct in social psychology. Social psychology: Handbook of basic principles.

[b8] Friedman R, Deci EL, Elliot AJ, Moller AC, Aarts H (2010). Motivational synchronicity: Priming motivational orientations with observations of others' behaviors. Motivation and Emotion.

[b9] Gaudreau P, Blondin JP (2002). Development of a questionnaire for the assessment of coping strategies employed by athletes in competitive sport settings. Psychology of Sport and Exercise.

[b9002] Gaudreau P, Carraro N, Miranda D (2012). From goal motivation to goal progress: the mediating role of coping in the Self-Concordance Model. Anxiety, Stress, and Coping.

[b10] Gauvin L, Rejeski WJ (1993). The Exercise-Induced Feeling Inventory—Development and initial validation. Journal of Sport & Exercise Psychology.

[b11] Gollwitzer PM, Sorrentino RM, Higgins ET (1990). Action phases and mind-sets. Handbook of motivation and cognition.

[b12] Goss P (1999). Close to the wind: An extraordinary story of triumph over adversity.

[b13] Haase CM, Poulin MJ, Heckhausen J (2012). Happiness as a motivator: Positive affect predicts primary control striving for career and educational goals. Personality and Social Psychology Bulletin.

[b14] Hayduk LA (1987). Structural equation modeling with LISREL.

[b15] Healy LC, Roberts R, Hardy J (2009).

[b16] Hodgins HS, Yacko HA, Gottlieb E (2006). Autonomy and nondefensiveness. Motivation and Emotion.

[b17] Johnson M (2011). Gold rush: What makes an Olympic champion?.

[b18] Klein HJ, Wesson MJ, Hollenbeck JR, Alge BJ (1999). Goal commitment and the goal-setting process: Conceptual clarification and empirical synthesis. Journal of Applied Psychology.

[b9003] Koestner R, Lekes N, Powers TA, Chicoine E (2002). Attaining personal goals: Self-concordance plus implementation intentions equals success. Journal of Personality and Social Psychology.

[b9004] Koestner R, Otis N, Powers TA, Pelletier L, Gagnon H (2008). Autonomous motivation, controlled motivation, and goal progress. Journal of Personality.

[b19] Lazarus RS (1991). Emotion and adaptation.

[b20] Lazarus RS, Folkman S (1984). Stress appraisal and coping.

[b21] Levesque C, Copeland KJ, Sutcliffe RA (2008). Conscious and nonconscious processes: Implications for self-determination theory. Canadian Psychology-Psychologie Canadienne.

[b22] Locke EA, Latham GP (1990). A theory of goal setting and task performance.

[b23] Locke EA, Latham GP (2002). Building a practically useful theory of goal setting and task motivation—A 35-year odyssey. American Psychologist.

[b24] Louro MJ, Pieters R, Zeelenberg M (2007). Dynamics of multiple-goal pursuit. Journal of Personality and Social Psychology.

[b25] Masicampo EJ, Baumeister RF (2011a). Consider it done! Plan making can eliminate the cognitive effects of unfulfilled goals. Journal of Personality and Social Psychology.

[b26] Masicampo EJ, Baumeister RF (2011b). Unfulfilled goals interfere with tasks that require executive functions. Journal of Experimental Social Psychology.

[b27] McGregor HA, Elliot AJ (2002). Achievement goals as predictors of achievement-relevant processes prior to task engagement. Journal of Educational Psychology.

[b28] Moller AC, Deci EL, Ryan RM (2006). Choice and ego-depletion: The moderating role of autonomy. Personality and Social Psychology Bulletin.

[b29] Ntoumanis N, Edmunds J, Duda JL (2009). Understanding the coping process from a self-determination theory perspective. British Journal of Health Psychology.

[b30] Preacher KJ, Hayes AF (2008). Asymptotic and resampling strategies for assessing and comparing indirect effects in multiple mediator models. Behavior Research Methods.

[b31] Radel R, Sarrazin P, Pelletier L (2009). Evidence of subliminally primed motivational orientations: The effects of unconscious motivational processes on the performance of a new motor task. Journal of Sport & Exercise Psychology.

[b32] Ratelle CF, Baldwin MW, Vallerand RJ (2005). On the cued activation of situational motivation. Journal of Experimental Social Psychology.

[b9005] Ryan RM, Deci EL (2000). Self-determination theory and the facilitation of intrinsic motivation, social development, and well-being. American Psychologist.

[b9006] Ryan RM, Koestner R, Deci EL (1991). Ego-involved persistence: When free-choice behavior is not intrinsically motivated. Motivation and Emotion.

[b33] Sheldon KM, Gardener W, Shah J (2008). The interface of motivational science and personology: Self-concordance, quality motivation, and multi-level personality integration. Handbook of motivational science.

[b34] Sheldon KM, Elliot AJ (1998). Not all personal goals are personal: Comparing autonomous and controlled reasons for goals as predictors of effort and attainment. Personality and Social Psychology Bulletin.

[b35] Sheldon KM, Elliot AJ (1999). Goal striving, need satisfaction, and longitudinal well-being: The self-concordance model. Journal of Personality and Social Psychology.

[b9007] Sheldon KM, Houser-Marko L (2001). Self-concordance, goal attainment, and the pursuit of happiness: Can there be an upward spiral?. Journal of Personality and Social Psychology.

[b36] Skinner EA, Ryan RM, Edge K, Deci EL (2002). Self-determination, coping, and development. Self-determination theory: Extensions and applications.

[b37] Smith A, Ntoumanis N, Duda J (2007). Goal striving, goal attainment, and well-being: Adapting and testing the self-concordance model in sport. Journal of Sport & Exercise Psychology.

[b9008] Smith A, Ntoumanis N, Duda J (2010). An investigation of coach behaviors, goal motives, and implementation intentions as predictors of well-being in sport. Journal of Applied Sport Psychology.

[b38] Smith A, Ntoumanis N, Duda JL, Vansteenkiste M (2011). Goal striving, coping, and well-being: A prospective investigation of the self-concordance model in sport. Journal of Sport & Exercise Psychology.

[b39] Strack F, Schwarz N, Bless H, Kubler A, Wanke M (1993). Awareness of the influence as a determinant of assimilation versus contrast. European Journal of Social Psychology.

[b40] Weinberg RS, Burke KL, Jackson A (1997). Coaches' and players' perceptions of goal setting in junior tennis: An exploratory investigation. Sport Psychologist.

[b41] Williamson JW, Fadel PJ, Mitchell JH (2006). New insights into central cardiovascular control during exercise in humans: A central command update. Experimental Physiology.

[b48] Wrosch C, Miller GE, Scheier MF, Pontet SB (2007). Giving up on unattainable goals: Benefits for health?. Personality and Social Psychology Bulletin.

[b42] Zimmerman BJ, Pintrich P, Paulsen AS (1995). Self-monitoring during collegiate studying: An invaluable tool for academic self-regulation. New directions in college teaching and learning: Understanding self-regulated learning.

